# Supplementary material for: COVID-19 pandemic and risk factor measurement in individuals with cardio-renal-metabolic diseases: A retrospective study in the United Kingdom
Source: PLoS One. 2025 Apr 24;20(4):e0319438. doi: 10.1371/journal.pone.0319438 (PMC12021215; doi:10.1371/journal.pone.0319438)
Supplement: S1 Table — (PDF) [file pone.0319438.s001.pdf]

S1 Table. Target measurements for defining controlled risk factors

Variables	Target value
HbA1c (%)	<7.5
BMI (kg/m^2)	<30
Blood pressure, Systolic (mmHg)	<140
Blood pressure, Diastolic (mmHg)	<90
Total cholesterol (mmol/L)	<5
Smoking	Ex or Non-smoker
