# Supplementary material for: COVID-19 pandemic and risk factor measurement in individuals with cardio-renal-metabolic diseases: A retrospective study in the United Kingdom
Source: PLoS One. 2025 Apr 24;20(4):e0319438. doi: 10.1371/journal.pone.0319438 (PMC12021215; doi:10.1371/journal.pone.0319438)
Supplement: S2 Table — (PDF) [file pone.0319438.s002.pdf]

S2 Table. Follow-up duration and missing values for baseline risk factors

Characteristics	T2DM sub-cohort			CVD sub-cohort			CKD sub-cohort		
	Eligible cohort on 1st March 2018	Eligible cohort on 1st March 2019	Eligible cohort on 1st March 2020	Eligible cohort on 1st March 2018	Eligible cohort on 1st March 2019	Eligible cohort on 1st March 2020	Eligible cohort on 1st March 2018	Eligible cohort on 1st March 2019	Eligible cohort on 1st March 2020
Follow-up duration (years), mean (SD)	0.9 (0.3)	0.9 (0.3)	0.8 (0.3)	0.9 (0.3)	0.8 (0.3)	0.8 (0.3)	0.9 (0.2)	0.8 (0.3)	0.7 (0.3)
Follow-up duration (years), median [IQR]	1.0 [1.0,1.0]	1.0 [0.8,1.0]	1.0 [0.5,1.0]	1.0 [1.0,1.0]	1.0 [0.7,1.0]	1.0 [0.5,1.0]	1.0 [1.0,1.0]	1.0 [0.7,1.0]	1.0 [0.5,1.0]
Total follow-up duration (years)	52344.9	44424.2	30605.7	35251.8	27099.7	14936.0	43630.4	36000.4	23741.5
**Number of patients with missing values**									
Blood pressure (mmHg), median [IQR]	10876	9449	7707	7246	6338	5005	6024	4925	3188
HbA1c at baseline (%), median [IQR]	16179	13260	9932						
BMI (kg/m^2), mean (SD)	20756	18094	13765	24549	20342	14200	20193	16215	9772
Total cholesterol (mmol/L), median [IQR]	17006	14679	11555	18679	15980	11555	16355	13418	8224
LDL (mmol/L), median [IQR]	21342	17494	13920	21755	18011	13419	18703	15128	9546
HDL (mmol/L), median [IQR]	18683	15918	12254	19864	16897	12240	17508	14351	8815
