# Supplementary material for: COVID-19 pandemic and risk factor measurement in individuals with cardio-renal-metabolic diseases: A retrospective study in the United Kingdom
Source: PLoS One. 2025 Apr 24;20(4):e0319438. doi: 10.1371/journal.pone.0319438 (PMC12021215; doi:10.1371/journal.pone.0319438)
Supplement: S3 Table — (PDF) [file pone.0319438.s003.pdf]

S3 Table. Unadjusted odds ratios for having at least one record of all risk factors for individuals in the T2DM, CVD, and CKD cohort during the pre-pandemic period of 2018-2019 and 2019-2020, and the pandemic period of 2020-2021

**Variables**	**2018**		**2019**		**2020**		**Cohort**
	Events/Total Patients	OR (95%CI)	Events/Total Patients	OR (95%CI)	Events/Total Patients	OR (95%CI)	
**Age (years)**	22,214/55,622		18,529/48,363		5,597/35,806		T2DM
18-44		Reference		Reference		Reference	
45-64		3.58 (3.32, 3.87)		3.80 (3.48, 4.15)		3.00 (2.59, 3.48)	
65-74		5.39 (4.98, 5.83)		5.39 (4.93, 5.89)		3.98 (3.43, 4.63)	
≥75		4.29 (3.97, 4.64)		4.41 (4.04, 4.82)		3.17 (2.72, 3.68)	
**Sex**							
Female		Reference		Reference		Reference	
Male		1.49 (1.44, 1.54)		1.47 (1.41, 1.52)		1.39 (1.31, 1.47)	
**Ethnicity**							
White		Reference		Reference		Reference	
Black		0.79 (0.72, 0.87)		0.76 (0.68, 0.85)		0.80 (0.67, 0.97)	
Mixed/Other		0.95 (0.87, 1.03)		0.93 (0.85, 1.02)		0.91 (0.79, 1.05)	
South Asian		0.92 (0.86, 0.99)		1.02 (0.94, 1.11)		1.04 (0.93, 1.17)	
**IMD (quintiles)**							
1		Reference		Reference		Reference	
2		1.02 (0.96, 1.07)		1.16 (1.09, 1.23)		1.15 (1.04, 1.26)	
3		1.02 (0.97, 1.08)		1.26 (1.19, 1.33)		1.15 (1.05, 1.26)	
4		0.97 (0.92, 1.02)		1.35 (1.27, 1.43)		1.23 (1.12, 1.35)	
5		1.14 (1.08, 1.20)		1.57 (1.48, 1.66)		1.33 (1.22, 1.46)	
**Comorbidities (Yes vs. No)**							
**CKD**		1.28 (1.23, 1.33)		1.20 (1.15, 1.26)		1.13 (1.06, 1.22)	
**CVD**		1.35 (1.30, 1.40)		1.32 (1.27, 1.38)		1.19 (1.11, 1.26)	
**Hypertension**		2.25 (2.17, 2.34)		2.01 (1.93, 2.09)		1.97 (1.85, 2.10)	
**Age (years)**	14,834/48,685		11,822/42,045		3,109/29,641		CVD
18-44		Reference		Reference		Reference	

45-64		3.10 (2.60, 3.68)		2.73 (2.25, 3.31)		2.43 (1.69, 3.49)	
65-74		4.12 (3.47, 4.90)		3.43 (2.83, 4.16)		3.13 (2.18, 4.49)	
≥75		2.93 (2.47, 3.48)		2.46 (2.03, 2.98)		2.21 (1.55, 3.17)	
**Sex**							
Female		Reference		Reference		Reference	
Male		1.30 (1.25, 1.35)		1.29 (1.24, 1.35)		1.28 (1.19, 1.38)	
**Ethnicity**							
White		Reference		Reference		Reference	
Black		0.78 (0.65, 0.93)		0.87 (0.72, 1.07)		1.01 (0.71, 1.42)	
Mixed/Other		0.94 (0.83, 1.07)		0.90 (0.78, 1.04)		1.20 (0.96, 1.50)	
South Asian		1.39 (1.24, 1.55)		1.40 (1.23, 1.59)		1.58 (1.32, 1.90)	
**IMD (quintiles)**							
1		Reference		Reference		Reference	
2		0.99 (0.93, 1.05)		1.14 (1.06, 1.21)		1.34 (1.18, 1.53)	
3		1.00 (0.95, 1.06)		1.26 (1.18, 1.35)		1.52 (1.34, 1.72)	
4		1.04 (0.98, 1.11)		1.49 (1.39, 1.59)		1.82 (1.61, 2.07)	
5		1.44 (1.36, 1.53)		1.95 (1.82, 2.08)		2.27 (2.02, 2.57)	
**Comorbidities (Yes vs. No)**							
**CKD**		1.05 (1.01, 1.10)		1.02 (0.97, 1.07)		1.04 (0.96, 1.13)	
**T2DM**		2.82 (2.71, 2.94)		2.99 (2.86, 3.12)		2.89 (2.68, 3.12)	
**Hypertension**		1.57 (1.50, 1.64)		1.53 (1.45, 1.61)		1.77 (1.60, 1.95)	
**Age (years)**							
18-44		Reference		Reference		Reference	
45-64		1.92 (1.42, 2.58)		1.89 (1.33, 2.69)		1.14 (0.63, 2.04)	
65-74		2.84 (2.12, 3.82)		2.63 (1.86, 3.74)		1.66 (0.93, 2.94)	
≥75		2.11 (1.57, 2.82)		1.99 (1.41, 2.82)		1.15 (0.65, 2.03)	
**Sex**	10,251/37,637		7,652/31,147		1,889/19,377		CKD
Female		Reference		Reference		Reference	
Male		1.35 (1.29, 1.41)		1.40 (1.33, 1.47)		1.46 (1.33, 1.61)	
**Ethnicity**							
White		Reference		Reference		Reference	
Black		0.95 (0.82, 1.10)		1.00 (0.84, 1.20)		0.91 (0.65, 1.29)	

Mixed/Other		1.08 (0.92, 1.27)		1.14 (0.95, 1.36)		1.28 (0.95, 1.73)	
South Asian		1.46 (1.26, 1.69)		1.57 (1.32, 1.87)		1.51 (1.15, 1.98)	
**IMD (quintiles)**							
1		Reference		Reference		Reference	
2		0.97 (0.91, 1.03)		1.20 (1.11, 1.30)		1.27 (1.09, 1.48)	
3		0.95 (0.89, 1.02)		1.32 (1.22, 1.43)		1.44 (1.24, 1.67)	
4		1.01 (0.94, 1.08)		1.45 (1.33, 1.57)		1.56 (1.33, 1.82)	
5		1.35 (1.25, 1.45)		2.02 (1.86, 2.20)		1.87 (1.61, 2.18)	
**Comorbidities (Yes vs. No)**							
**CVD**		1.37 (1.31, 1.44)		1.39 (1.32, 1.47)		1.21 (1.10, 1.33)	
**T2DM**		3.56 (3.39, 3.73)		3.82 (3.61, 4.03)		3.40 (3.08, 3.75)	
**Hypertension**		1.68 (1.58, 1.79)		1.62 (1.51, 1.74)		1.77 (1.54, 2.04)	

Complete case univariable logistic regression models were used to estimate the association between each patient characteristic (age, sex, ethnicity, deprivation, and prevalent comorbidity (T2DM, CVD, CKD, and hypertension)) with the binary outcome variable of a patient having all the selected risk factors (systolic (SBP) and diastolic (DBP) blood pressure (BP), total cholesterol (TC), high-density lipoproteins (HDL), low-density lipoproteins (LDL), body mass index (BMI), smoking status, and HbA1c (for the T2DM sub-cohort only)) checked during a given period.

OR=odds ratio. IMD=indices of multiple deprivation.
