# Supplementary material for: COVID-19 pandemic and risk factor measurement in individuals with cardio-renal-metabolic diseases: A retrospective study in the United Kingdom
Source: PLoS One. 2025 Apr 24;20(4):e0319438. doi: 10.1371/journal.pone.0319438 (PMC12021215; doi:10.1371/journal.pone.0319438)
Supplement: S1 Fig — (PDF) [file pone.0319438.s004.pdf]

S1 Fig. Cohort definition sample diagram

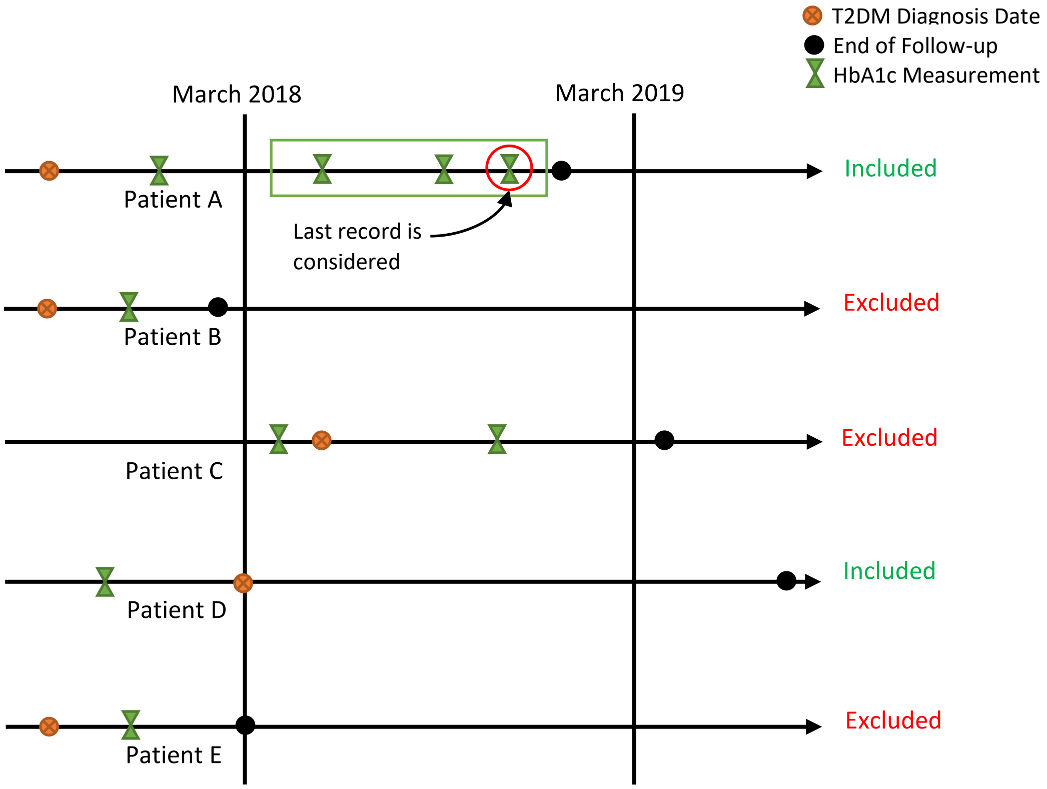

Patient A and Patient D are included
Patient A has ≥1 HbA1c record within 2018-2019
Patient D has no HbA1c record within 2018-2019
Overall, among those who are included, 50% of them had at least ≥1 record
