# Supplementary material for: COVID-19 pandemic and risk factor measurement in individuals with cardio-renal-metabolic diseases: A retrospective study in the United Kingdom
Source: PLoS One. 2025 Apr 24;20(4):e0319438. doi: 10.1371/journal.pone.0319438 (PMC12021215; doi:10.1371/journal.pone.0319438)
Supplement: S2 Fig — (PDF) [file pone.0319438.s005.pdf]

S2 Fig. Study population flow diagram

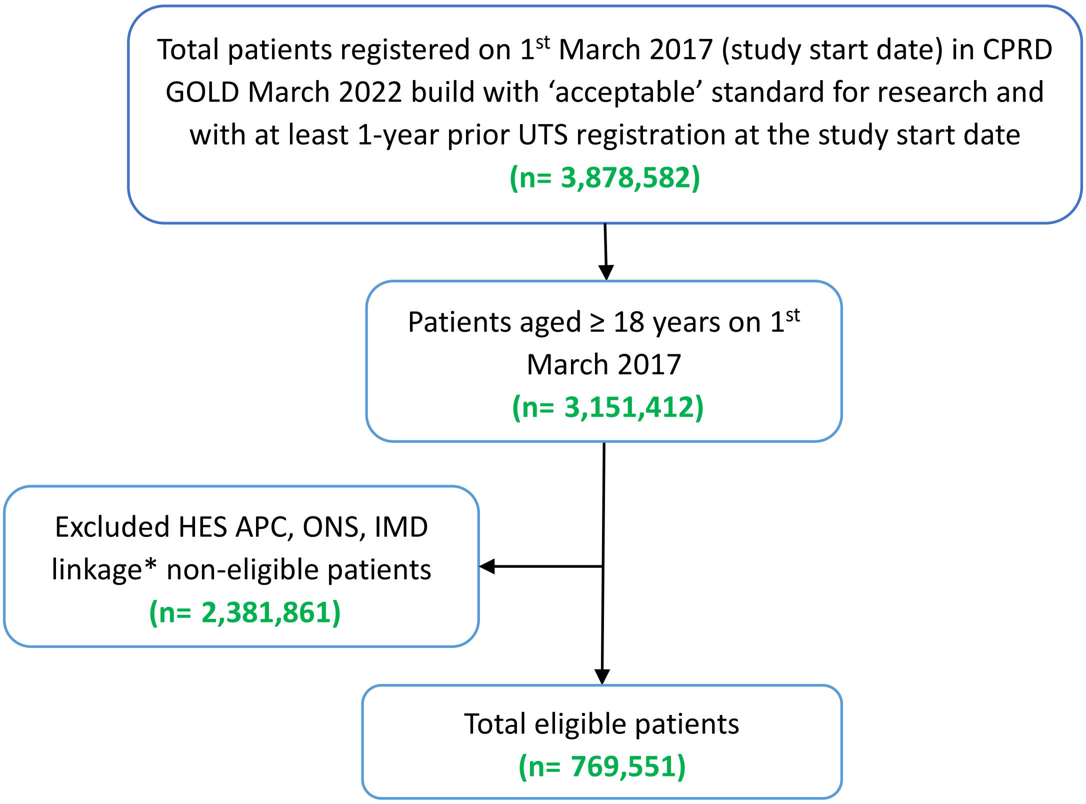

CPRD: Clinical Practice Research Datalink; UTS: Up-to-standard; HES APC: Hospital Episode Statistics Admitted Patient Care; ONS: Office for National Statistics; IMD: Index of Multiple Deprivation.
*HES and ONS linkage were available only up to March 2021.
