# Supplementary material for: COVID-19 pandemic and risk factor measurement in individuals with cardio-renal-metabolic diseases: A retrospective study in the United Kingdom
Source: PLoS One. 2025 Apr 24;20(4):e0319438. doi: 10.1371/journal.pone.0319438 (PMC12021215; doi:10.1371/journal.pone.0319438)

S4 Fig. Proportion of individuals, by sex, in the T2DM, CVD and CKD cohort who had at least one measurement of the selected risk factors recorded in CPRD during the pre-pandemic period of 2018-2019 and 2019-2020, and the pandemic period of 2020-2021

a) T2DM Cohort

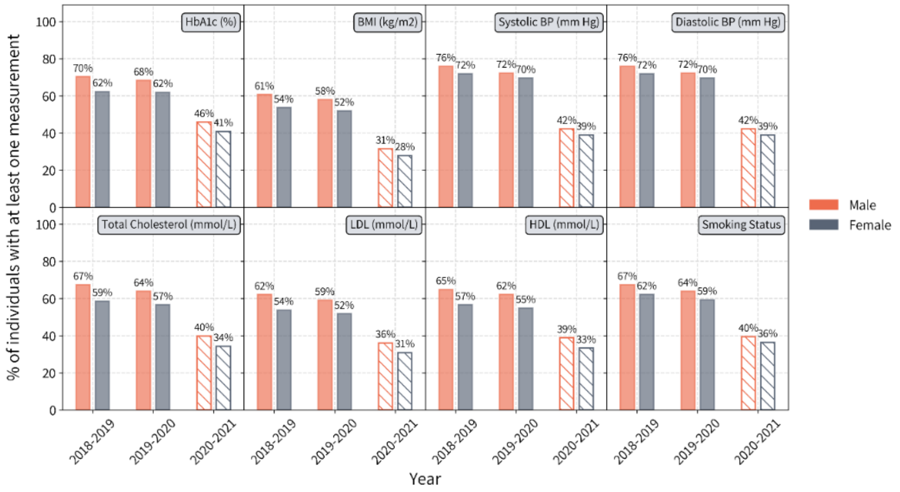

b) CVD Cohort

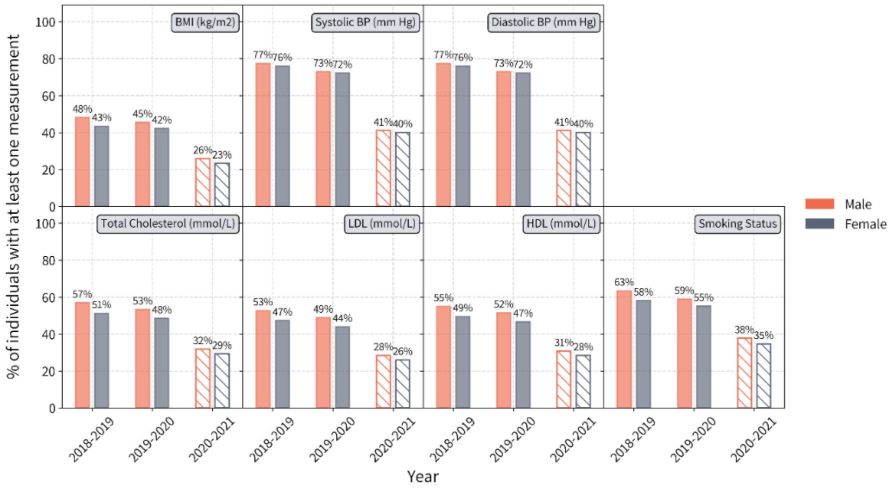

c) CKD Cohort

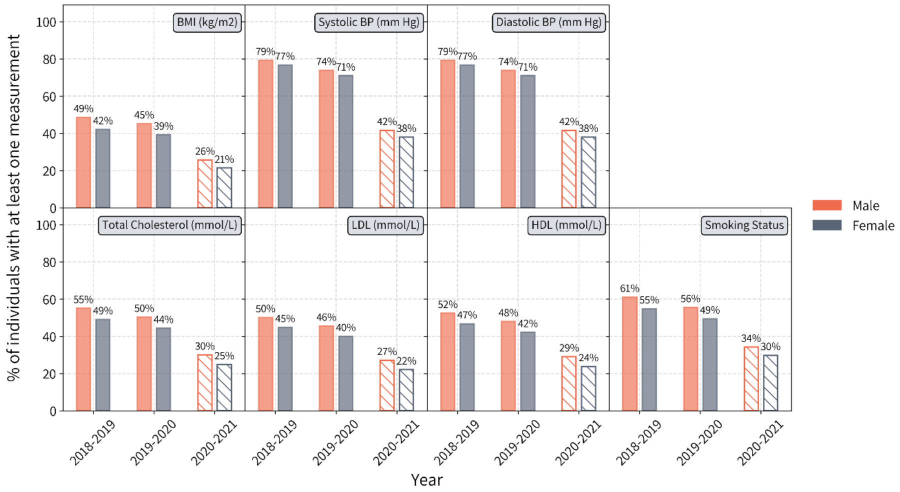

Supplement: S4 Fig — (PDF) [file pone.0319438.s007.pdf]
