# Supplementary material for: COVID-19 pandemic and risk factor measurement in individuals with cardio-renal-metabolic diseases: A retrospective study in the United Kingdom
Source: PLoS One. 2025 Apr 24;20(4):e0319438. doi: 10.1371/journal.pone.0319438 (PMC12021215; doi:10.1371/journal.pone.0319438)

S7 Fig. Proportion of individuals in the restricted (to those with complete follow-up) T2DM, CVD and CKD cohort who had at least one measurement of the selected risk factors recorded in CPRD during the pre-pandemic period of 2018-2019 and 2019-2020, and the pandemic period of 2020-2021

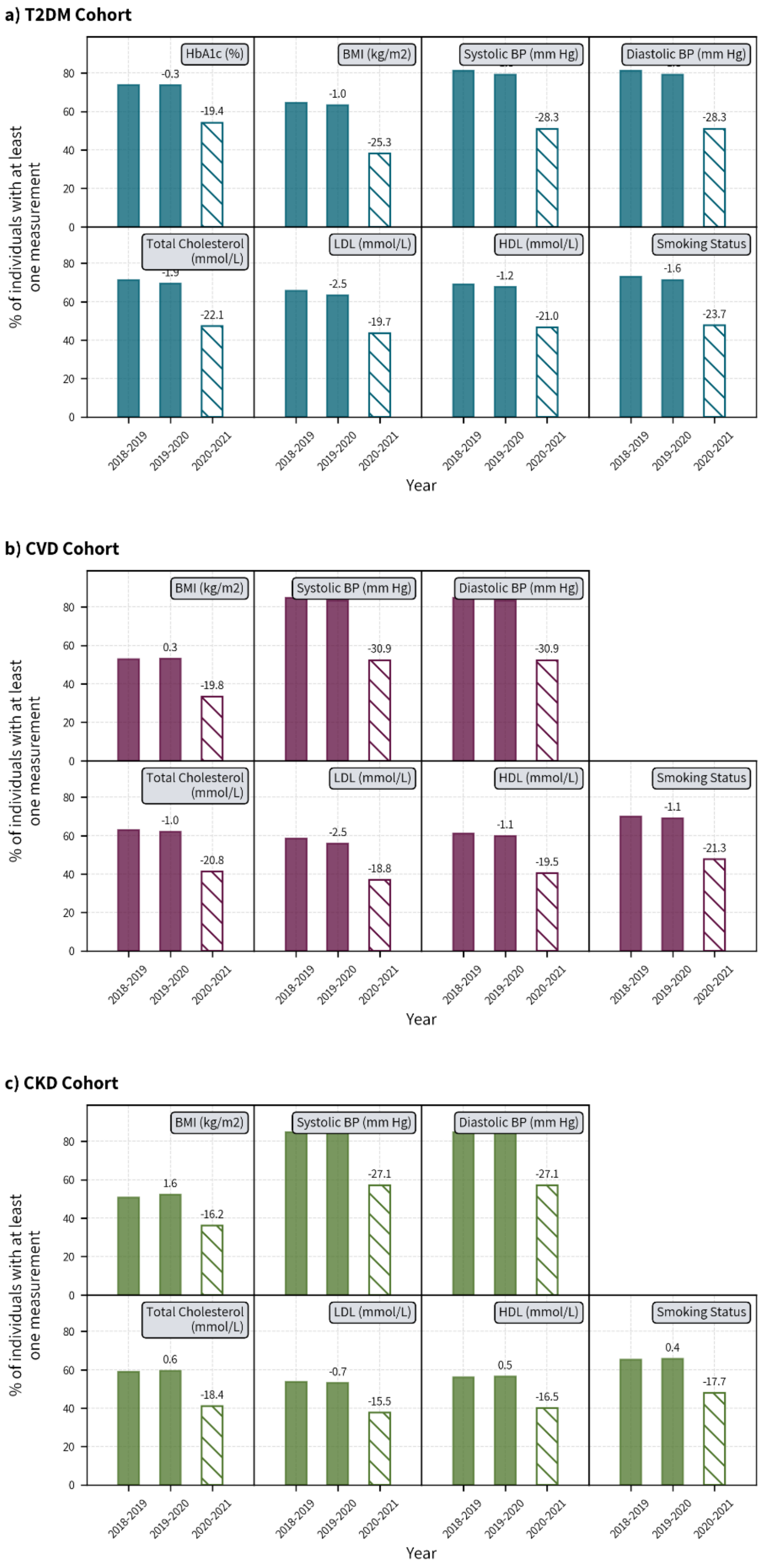

Supplement: S7 Fig — (PDF) [file pone.0319438.s010.pdf]
