# Supplementary material for: COVID-19 pandemic and risk factor measurement in individuals with cardio-renal-metabolic diseases: A retrospective study in the United Kingdom
Source: PLoS One. 2025 Apr 24;20(4):e0319438. doi: 10.1371/journal.pone.0319438 (PMC12021215; doi:10.1371/journal.pone.0319438)

S11 Fig. Mean recorded values of selected risk factors among those with at least one measurement in CPRD in the T2DM, CVD, and CKD cohort during the pre-pandemic period of 2018-2019 and 2019-2020, and the pandemic period of 2020-2021, by deprivation

a) T2DM Cohort

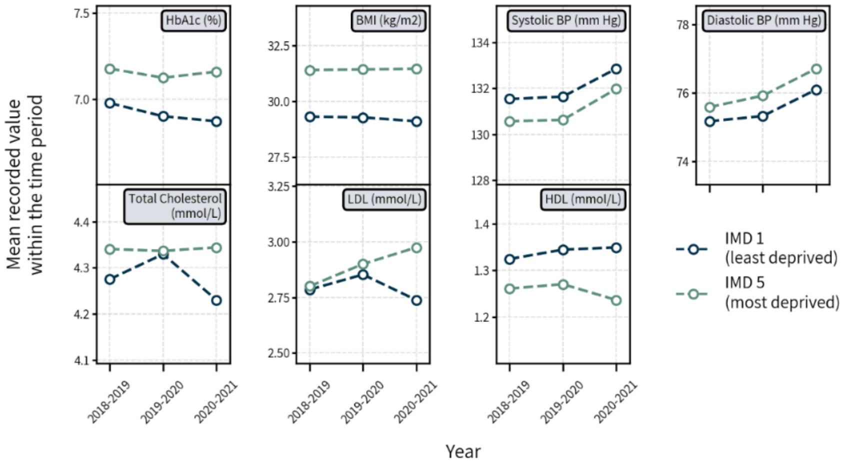

b) CVD Cohort

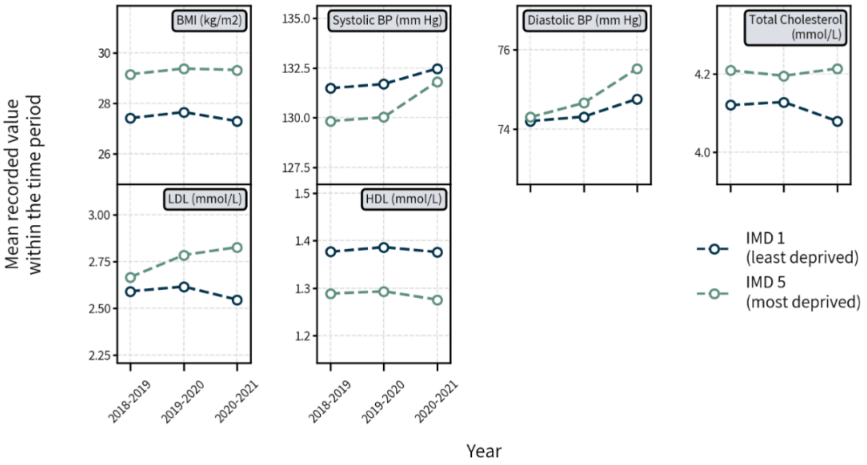

c) CKD Cohort

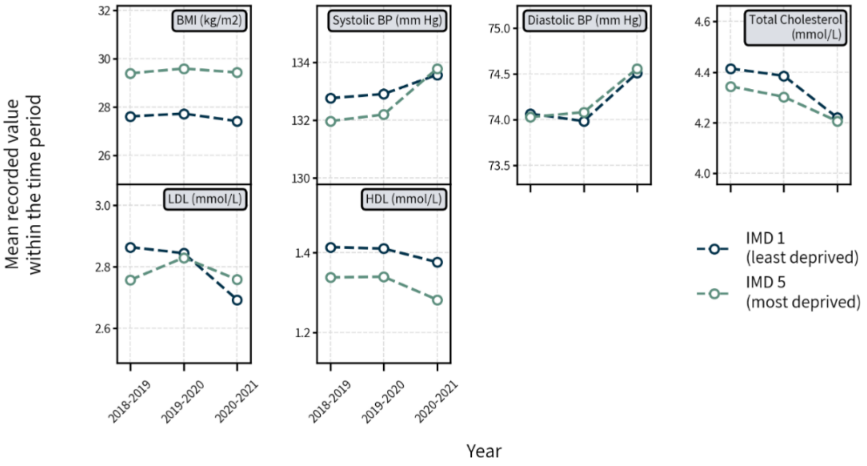

Supplement: S11 Fig — (PDF) [file pone.0319438.s014.pdf]
