# Supplementary material for: COVID-19 pandemic and risk factor measurement in individuals with cardio-renal-metabolic diseases: A retrospective study in the United Kingdom
Source: PLoS One. 2025 Apr 24;20(4):e0319438. doi: 10.1371/journal.pone.0319438 (PMC12021215; doi:10.1371/journal.pone.0319438)
Supplement: S1 Checklist — Statements (RECORD Checklist). (PDF) [file pone.0319438.s020.pdf]

S1 Statements (RECORD Checklist)

Participants (RECORD 6.2): The codes used for this research were obtained using CPRD Code Browser and were validated against code lists used in previous studies using the same datasets.

Data access and cleaning methods (RECORD 12.1): Authors ShS, FZ, KK, and CG had full access to the datasets used in this research. ShS performed the data cleaning and analysis; FZ, KK, and CG supervised the data cleaning and analysis.

Linkage (RECORD 12.3): Access to linked data is subject to CPRD protocol approval by ISAC. Authors are required to clearly state which datasets they require linkage to in the protocol. To receive the data, they are requested to submit a linkage request form to ISAC that requires additional approval. Data linkage is carried out by the Trusted Third Party NHS Digital. For further information refer to: https://www.cprd.com/linked-data
